# A combined theory-based explanatory model for predicting tourists’ travel intentions during the COVID-19 pandemic: the role of individual’s assessment of the compliance with non-pharmaceutical interventions

**DOI:** 10.1007/s44202-022-00046-2

**Published:** 2022-09-20

**Authors:** Vu Thi Thao, Andreas Philippe Hüsser, Timo Ohnmacht

**Affiliations:** Competence Center for Mobility, Lucerne University of Applied Sciences and Arts, Rösslimatte 48, P.O. Box 2960, CH-6002 Lucerne, Switzerland

**Keywords:** COVID-19, Travel intentions, Health belief model, Theory of planned behaviour, Risk-taking behaviour, NPIs

## Abstract

This study examines the impact of the COVID-19 pandemic and tourist’s assessments of non-pharmaceutical public-health interventions (NPIs) in relation to their travel intentions. It uses a combined theoretical model that incorporates the Domain-Specific Risk-Taking Scale (DOSPERT) in the recreational domain, the Health Belief Model (HBM) and the Theory of Planned Behaviour (TPB). A large-scale population study that is representative of Switzerland has been carried out to validate the model (N = 1683; 39% response rate). We use a regression model based on mean indices for our explanatory model. Health beliefs, namely perceived susceptibility and severity, are important predictors of travel intentions. The perceived benefits of and barriers to compliance with NPIs when travelling also have a substantial influence on travel intentions. The results demonstrated that the factors of the HBM tend to have a stronger influence than other significant factors, such as the perceived behavioural control of the TPB. As a situational context, the ability to work from home increases the intention to travel. The achievement of the present research is a validated empirical theory-based model that is suitable for practical and managerial implications. It can be used to create and evaluate measures and interventions that address the social psychological influencing factors.

## Introduction

The world has just experienced one of the worst global health and economic crises of the last century [42]. By February 8, 2022, the coronavirus disease (COVID-19) had infected more than 397 million people worldwide and contributed to 5.75 million deaths [28]. The COVID-19 pandemic caused a 3.5-percent contraction in global economic growth in 2020 [64]. It has also severely affected the tourism sector. In 2020, the number international overnight visitors dropped 74% compared to 2019 [58]. In Switzerland, the willingness to take overnight trips of the Swiss population declined from roughly 90% in 2019 to 55% in 2021 [56]. Touristic day trips of the Swiss were reduced by nearly a third between 2018 and 2020 [20]. There was a decline of 70% in international overnight visitors comparing the years 2020 to 2019 for January to August [19]. This drop in touristic demand is likely due to a combination of legal restrictions and a ‘voluntary’ decline in demand to protect one’s health against contracting the virus. Consequently, Switzerland’s tourism collapsed in 2020 [32]. It caused a major impact on tourist service providers, such as travel agencies and tour operators who have had a loss in sales of nearly 70% from 2019 to 2020 [25]. The Swiss tourism sector has recovered slowly mainly thanks to domestic travellers [32].

Vacations are believed to improve health and well-being, particularly when individuals are in situations of physical and psychological strain [18]. However, since the COVID-19 outbreak, tourists have had to incorporate the risks of being exposed to a virus into their decision-making regarding travelling. In a global pandemic, travel intentions are influenced by concerns about health and the risks of having trips cancelled and becoming stranded overseas or placed in quarantine [59]. This risk can be reduced through compliance with non-pharmaceutical interventions (NPIs), such as social distancing, health-screening and testing, and the correct wearing of face masks [62]. This situation prompts an intriguing research question for this present paper: How does the individual’s assessment of the need for compliance with NPIs to mitigate the risk and impact of the COVID-19 pandemic influence their travel decisions? We seek to answer this research question by linking theoretical models. We adopt a theory-based explanatory model from the field of social psychology that blends one scale and two well-established theories for predicting the intention to travel. The Domain-Specific Risk-Taking Scale (DOSPERT) in the recreational domain, the Health Belief Model (HBM) [10] and the Theory of Planned Behaviour (TPB) will be used to predict tourists’ travel intentions [3, 48]. For an application on tourists’ intentions to implement protective measures see Ohnmacht et al. [43]. Our modelling approach aims to provide practical implications derived from the empirical findings, i.e., pointers for interventions.

Unlike the previous research that mainly concerns about travel avoidance (e.g., [12, 65]). we take into account individual assessments of compliance with NPIs against the transmission of coronavirus when travelling as a central element in the explanatory factors of the HBM and the TPB. Are these measures a hindrance to travel intentions, or do they promote travel by making people feel that travel is safe? The analysis is based on a nationally representative survey of 1,683 Swiss residents (39% response rate). The explanatory factors of these theories are used to predict travel intentions that are defined as a plan to perform a movement to places outside the tourists’ usual environment for personal reasons and to stay in their destinations for at least one night (e.g., [57]).

The remainder of this paper is organised as follows. The literature review section summarizes recent tourism studies on the impacts of the COVID-19 pandemic that have adopted risk perceptions to predict travel intentions and develops the hypotheses and the theory-based explanatory model for the current study. The methods section describes the data collection, measures, and data-analysis procedures as well as the modelling approach. The results section presents key findings of multivariate modelling. Finally, in the conclusion section, we discuss the theoretical and practical implications of our results to guide future research.

## Literature review, theories and hypotheses development

Various authors have investigated the impacts of the coronavirus pandemic on holiday travel intentions during the pandemic [7, 26, 45, 49]. However, some of the shortcomings in this stream of literature should be addressed. Firstly, most studies have tackled the topic from the perspective of a single theoretical model extended by the addition of one or more than two influencing factors stemming from other concepts [1, 49]. Secondly, while research has been focused on travel avoidance (e.g., [12, 65]), only a few studies have linked travel intentions to compliance with NPIs and its effects on travel intentions (e.g., [14, 44]). Thirdly, a majority of studies rely on non-probabilistic convenience samples or the random recruitment of study participants from the internet (e.g., social network platforms), usually conducted online (e.g., [1, 7, 26, 49]). Fourthly, while most of the literature can specify the factors that have influenced their research and provide implications for tourism practitioners, they do not elaborate on the policy dimension, such as recommendations for designing interventions (e.g., [7, 11, 26]).

Tourism is a leisure activity characterized by a voluntary form of behaviour [33], which enjoys much greater risk acceptance from individuals than involuntary activities, given the same benefit level [54]. Studies have adopted risk measures stemming from various theoretical backgrounds. Obviously, there is no consensus on the concept of risk: its definitions vary in different disciplines and study areas (e.g., [6]). For example, behavioural economists and experimental psychologists define risk objectively based on known probabilities of different monetary outcomes in gambles. This conceptualization is derived from the expected utility framework, where individuals are supposed to weigh different (monetary) outcomes by their corresponding (known) probabilities and choose the outcome with the highest expected utility (e.g., [29, 34]). An opposite conceptualization of risk is that of Slovic et al. [52]. They relied on the so-called ‘psychometric paradigm’, which uses multivariate statistical analysis to identify meaningful factors that can explain individual differences in risk perceptions and risk attitudes [52]. In this tradition of research, risk is conceptualized as a perceptual variable that is ‘inherently subjective’ ([53], p. 4). The authors argue that risk cannot be measured or quantified in an objective manner [53], as it is the case with a COVID-19 infection during vacations. In tourism, subjective perceived risks and their influences on tourist behaviour have been the focal of risk studies [63]. Against the background of our aim to provide pointers for interventions, three major and suitable approaches must be highlighted: the DOSPERT, the HBM and the TPB, which can be used to predict tourists’ travel intentions [3, 10, 48].

## Domain-specific risk-taking (DOSPERT) scale

The Domain-Specific Risk-Taking (DOSPERT) scale has been used widely as a psychological scale to measure the risk attitudes and behaviour of lay people in different domains across various population groups and cultures [51]. Developed by (see also Weber et al. [60]), the scale was derived primarily from the psychological risk-return models in which risk-taking is seen to result from an assessment of the benefits and consequences of the risk. The DOSPERT scale assesses risk attitudes in five domains, including leisure and recreational decisions. Respondents were asked to rate the level of the likelihood that they would be willing to undertake the described behaviour, e.g., camping in the wilderness. The higher the score on the risk-attitude scale the greater risk-taking intention [10]. Therefore, we hypothesize that:

H1: The greater the risk attitude in the general leisure and recreational domain, the greater the intention to travel to a tourist destination during the COVID-19 pandemic.

### Health belief model (HBM)

The perception of the health risks is one of the most important factors influencing tourist behaviour [26]. The HBM was adopted to predict travel intentions in several studies concerning the coronavirus pandemic [7, 26]. The model assumes that individuals’ preventive health behaviour is primarily explained by the perceived susceptibility and perceived severity of the negative health impacts of a disease, as well as the perceived benefits of and perceived barriers to taking action to avoid adverse consequences for their health [47]. Drawing upon these studies and our focus on the NPIs, we hypothesize that:

H2: The higher the perceived susceptibility to the coronavirus while travelling, the lower the intention to travel to a tourist destination during the COVID-19 pandemic.

H3: The higher the perceived severity of the coronavirus, the lower the intention to travel to a tourist destination during the COVID-19 pandemic.

H4: The higher the perceived benefits of complying with NPIs against the coronavirus while travelling, the greater the intention to travel to a tourist destination during the COVID-19 pandemic.

H5: The higher the perceived barriers to complying with NPIs against the coronavirus, the lower the intention to travel to a tourist destination during the COVID-19 pandemic.

To increase the explanatory power of the HBM, Rosenstock et al. [48] proposed incorporating self-efficacy into the original model. Self-efficacy is the ‘conviction that one can successfully execute the behaviour required to produce the outcomes’ ([48], p. 178), and it is essential in predicting and achieving a change in (health) behaviour [55]. In this context, it can be understood that tourists believe that, through their behaviour, they can block the spread of the coronavirus. The findings of some studies in the tourism context have shown that self-efficacy has a significant impact on tourists’ preventive behaviour during both the pandemic [38] and post-pandemic periods [66]. Hence, we hypothesize that:

H6: The higher the perceived self-efficacy to protect society from COVID-19, the greater the intention to travel to a tourist destination during the COVID-19 pandemic.

### Theory of planned behaviour (TPB)

The theory of planned behaviour (TPB) is one of the most widely used models in predicting behaviour [5], including travel behaviour [7, 31]. According to Ajzen [3], the individual’s intention is the immediate antecedent of behaviour. Intentions are determined by attitudes, subjective norms and perceived behavioural control. The results of several empirical studies on travel intentions in the context of the coronavirus pandemic confirm a positive association between these factors and behavioural intentions [7, 49, 50]. Therefore, the following hypotheses are proposed:

H7: The more positive one’s attitude towards NPIs against the coronavirus when travelling, the greater the intention to travel to a tourist destination during the COVID-19 pandemic.

H8: The stronger the subjective norm regarding compliance with NPIs against the coronavirus when travelling, the higher the intention to travel to a tourist destination during the COVID-19 pandemic.

H9: The higher the perceived behavioural control over compliance with NPIs against the coronavirus when travelling, the greater the intention to travel to a tourist destination during the COVID-19 pandemic.

**Fig. 1:**
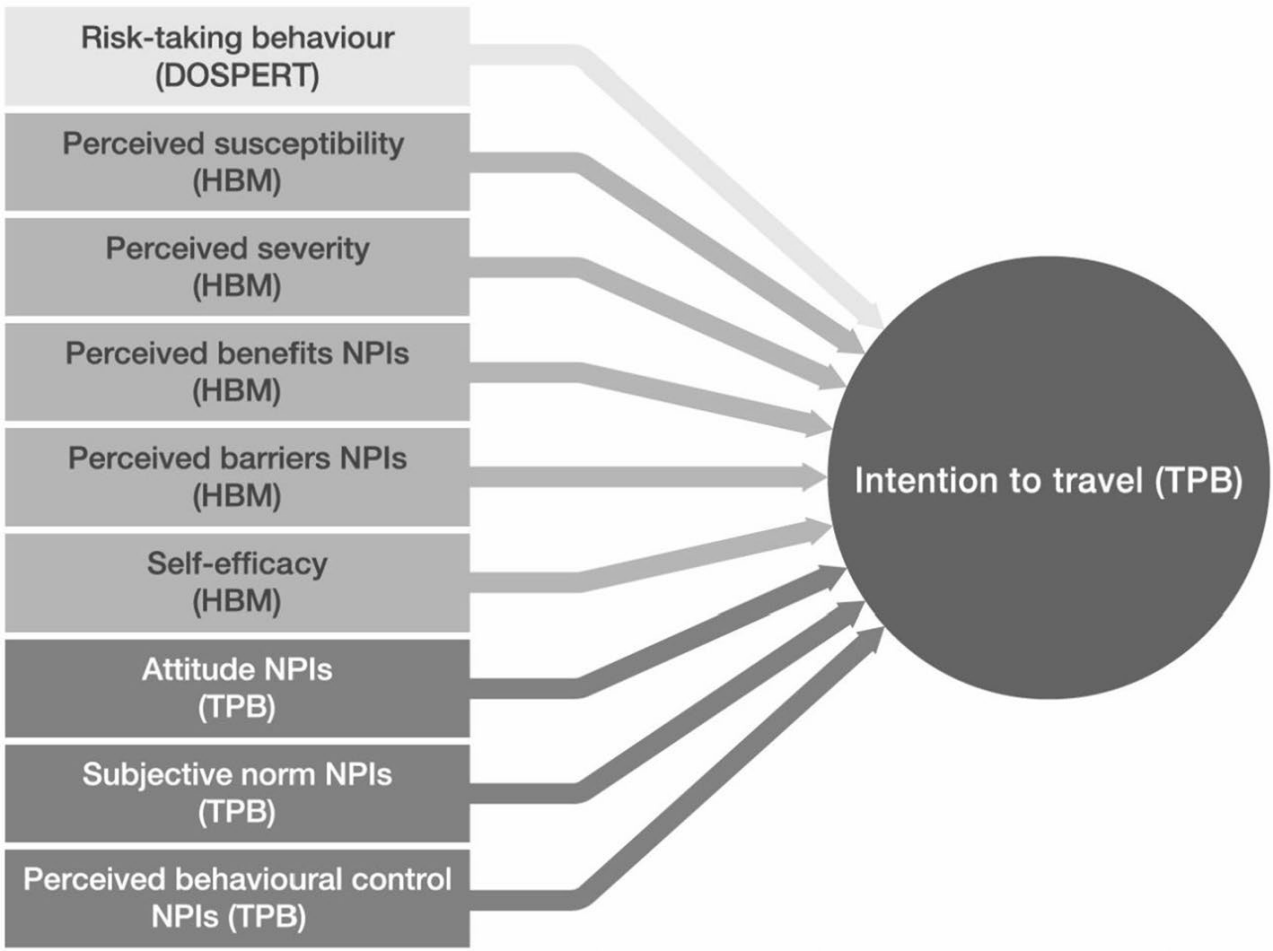
The conceptual model

To date, no study has combined the nine social psychological influencing factors of these two theories and the DOSPERT-scale into one combined theory-based explanatory model to predict tourists’ travel intentions. By identifying significant influences and the degree of their effects, measures to reduce the spread of the coronavirus in the tourism domain can be assessed and developed against the background of the tourists’ deliberative and cognitive decision-making processes. Figure 1 shows the conceptual model of this study, derived from the theories and the literature review. It depicts a combination of the DOSPERT scale, the HBM and the TPB to predict travel intentions to a touristic destination during the COVID-19 pandemic.

### Situational context: home-working possibilities

Besides individual assessments of the risk of the pandemic situation and the measures to be taken to decrease the risks, there are situational contexts that can be both a hindrance and a stimulant regarding touristic travel intentions during a pandemic. The coronavirus outbreak especially has turned working from home in a normal work arrangement for millions of workers worldwide [36]. As a special focus, we include an exemplary situational context for the possibility of working from home in the model presented in Fig. 1. Both the structural changes towards a service economy in Switzerland and the social changes due to the COVID-19 pandemic can be seen as accelerating the adoption of teleworking arrangements. According to the latest figures from the Swiss Federal Statistical Office (FSO), working from home increased more than six-fold between 2001 and 2020, rising from 250,000 to over 1.5 million employees and self-employed persons, equivalent to roughly a third of all Swiss employees. Moreover, the number of people spending 50% or more of their work time working from home increased to 12.8% of all employees [24]. Danalet et al. [13] estimated that 37.3% of the population will be working from home in Switzerland by 2030. The MOSAiCH survey (Measurement and Observation of Social Attitudes in Switzerland) showed that work situations changed for 83% of the respondents during the lockdown, with 26% of the respondents working entirely from home and 15% partly [16].

The situational context that can influence behaviour may be linked to theoretical considerations in environmental psychology known as ‘environmental contexts’ or ‘cues’ (built, technical, natural, social, cultural, economic, political). The theoretical explanation for the effect of working from home is rooted in discussions that such cultural, political or economic settings (environmental cues) strengthen or weaken the behavioural alternatives [35].

Linking the figures mentioned above to the tourism domain, employees who can work from home face fewer risks of job insecurity (pay reductions, job losses) due to having to quarantine after returning from their holidays. For instance, the measure of a quarantine on arrival may jeopardize the relationship between employers and employees. In fact, having to quarantine after returning from a holiday can increase job insecurity. Employees who can work from home face fewer problems at work from quarantining after returning from their holidays. Additionally, employed travellers may worry about not getting paid if they are quarantined [37]. This is clearly illustrated by a search question: “Do I get paid if I have to be in quarantine for 14 days?” conducted on October 13, 2021, that showed 237 million results on Google. The possibilities of working from home may increase the intention to travel during a pandemic. Thus, the following hypothesis is proposed:

H10: The possibility to work from home increases the intention to travel to a tourist destination during the COVID-19 pandemic.

Due to the novelty of our theoretical approach, only small and indirect links can be made to the previous research on hypothesis formulation. This is the reason we present related literature that is comparable to our empirical investigation. The hypothesis development is mainly based on our theory-based model.

## Method

### Sampling

The population in this study consisted of a representative cross-section of the Swiss population aged 18 years and over. Respondents were selected using stratified random sampling based on the three main linguistic regions (German and Romansh, French, and Italian) and gender (see Table 1). The sample frame is based on CASTEM (Sampling frame for drawing household samples from the census), the addresses of the respondents being obtained from the Swiss Federal Statistical Office.

**Table 1:** The sampling frame

Stratification		Sample	Response
Linguistic region	Gender	%	%
German and Romansh	Male	36	33
	Female	36	35
French	Male	12	12
	Female	12	15
Italian	Male	2	2
	Female	2	3
Total		100	100

Comparison between drawn sample and responses

Invitation letters containing a questionnaire booklet and a post-paid envelope were sent to 4530 potential respondents, who could also participate in the survey online by entering the personal code through the survey website’s link, which was accessible via a QR Code or by typing the URL address into the browser. Altogether, 164 addresses were reported as undeliverable for various reasons, such as residential relocations (sample-neutral losses). The survey was conducted between March and May 2021, that is, during the peak of the pandemic. To increase the response rate, reminder letters were sent to all the potential respondents two weeks later. In total, 1,683 respondents fully completed the survey, a response rate of approximately 39%. All responses were valid and were used in the anonymized data analysis.

### Demographic profile

As shown in Table 2, the socio-demographic characteristics of the sample prove to be highly representative when compared with the whole population [21, 22]. There were slightly more female respondents than their male counterparts. The average age was 54 years, with the age groups over 30 years accounting for almost 90%. About half of all participants had completed no more than compulsory schooling. According to the Swiss Federal Statistical Office, in 2020 about 34% of all Swiss employees worked from home at least once a month [23]. Since our survey was carried out during the peak of COVID-19, this figure is higher in our sample (44%), reflecting the figures in the Swiss MOSAiCH survey during the pandemic [16].

**Table 2:** Demographic profile of respondents

	Sample (%)	Swiss census 2020 (%)
Gender		
Male	48	50
Female	52	50
Age		
18–30	11	19
31–55	38	44
56–65	21	16
66 +	28	21
Education level		
Compulsory school	47	46
Upper secondary school	8	9
Professional (tertiary) education	20	15
Higher education	25	30
Working from home		
Yes	44	34
No	56	66

### Measures

The data used in this paper came from the first part of a survey that aimed to identify relevant factors influencing the behavioural intentions of Swiss tourists in respect of their planned travel behaviour and their intentions to comply with NPIs. The questionnaire was designed to cover all the variables in Fig. 1, as well as demographic information and information on home-working possibilities. The questionnaire was first composed in German, then translated into French and Italian to cover all three main language regions of Switzerland. The variables were measured by individual ratings using five-point Likert scales (e.g., 1: disagree strongly, 5: agree strongly) or semantic differentials (for the attitude measure). For risk behaviour during recreation, three relevant items were taken from DOSPERT [10]. The five variables of the HBM and the three variables of the TPB were each taken from previous studies and adapted to the survey goals (e.g., [31]). To test the reliability and validity of the scales, two pretests each with 300 individuals were carried out. The operationalization of the latent constructs and their corresponding items is presented in Table 3.

**Table 3:** Exploratory factor analysis to check factorial validity

Factor	Scale items	Loading	Eigenvalue	% of variance	Reliability α	Mean	SD	n
1	Risk-taking behaviour		1.282	1.989	0.751	1.91	1.06	1659
	rtb_1: Would you stay in a tent out in the wild, far removed from any town or campsite?	0.631						
	rtb_2: Would you join a white-water rafting tour in fast-flowing rivers in the spring?	0.784						
	rtb_3: Would you do risky sports (e.g., rock climbing, skydiving, etc.) regularly?	0.783						
2	Perceived susceptibility		2.283	4.271	0.904	3.54	1.10	1659
	sus_1: It’s likely that I will be exposed to the coronavirus when travelling at this time	0.702						
	sus_3: There is currently a high risk of infection from the coronavirus when travelling	0.870						
	sus_4: There is currently a high risk of passing on the coronavirus when travelling	0.812						
	sus_5: There is currently a high risk of coming into contact with the coronavirus when travelling	0.944						
3	Perceived severity		1.895	3.438	0.869	3.27	1.13	1664
	sev_1: Getting infected with the coronavirus would have severe consequences for my social life (friends, club, sport)	0.621						
	sev_2: Getting infected with the coronavirus would have severe consequences for my physical health	0.769						
	sev_3: Getting infected with the coronavirus would have severe consequences for my mental well-being	0.913						
	sev_4: Getting infected with the coronavirus would have severe consequences for my mental ability to perform	0.830						
4	Perceived benefits NPIs		1.688	2.908	0.825	3.78	.91	1658
	ben_3: The protective measures (*) effectively contain the coronavirus when people travel	0.855						
	ben_4: The protective measures reduce the risk of infection when people travel	0.850						
	ben_5: The protective measures make me feel safe when I travel	0.715						
	ben_6: By applying protective measures while travelling, I am behaving responsibly	0.451						
5	Perceived barriers NPIs		1.858	3.264	0.817	2.98	1.13	1659
	bar_1: For me, the costs (time, comfort, money) of applying protective measures when travelling are greater than the benefits	− 0.739						
	bar_2: For me, the effort of applying protective measures when travelling is greater than the benefits	− 0.769						
	bar_3: The protective measures are disturbing when travelling	− 0.692						
	bar_4: The protective measures prevent pleasant travelling	− 0.725						
6	Self-efficacy		1.627	2.693	0.819	4.02	0.90	1666
	se_1: With my behaviour, I can help to keep infection rates from increasing further during the pandemic	0.657						
	se_2: I can contribute to ending the pandemic soon	0.758						
	se_3: I can help protect society from the coronavirus	0.843						
	se_4: Risk groups are best protected if I apply the measures	0.585						
7	Attitude NPIs		14.093	30.015	0.958	4.27	0.90	1657
	att_npi_1: I find applying the coronavirus protective measures when travelling to be … bad–good	0.803						
	att_npi_2: … useless–useful	0.863						
	att_npi_3: … undesirable–desirable	0.723						
	att_npi_4: … inappropriate–appropriate	0.943						
	att_npi_6: … unimportant–important	0.892						
	att_npi_7: … not worthwhile–worthwhile	0.803						
	att_npi_8: … unnecessary–necessary	0.935						
	att_npi_9: … meaningless–meaningful	0.925						
8	Subjective norm NPIs		5.850	12.221	0.958	4.15	0.90	1644
	sno_npi_2: Most people who are important to me are in favour of applying protective measures when travelling	0.826						
	sno_npi_3: Most people who are important to me think that applying protective measures when travelling is a good idea	0.865						
	sno_npi_4: Most people who are important to me think I should apply protective measures when travelling	0.909						
	sno_npi_5: Most people who are important to me generally recommend applying protective measures when travelling	0.930						
	sno_npi_6: Most people who are important to me support me in applying protective measures when travelling	0.861						
	sno_npi_7: Most people who are important to me encourage me to apply protective measures when travelling	0.890						
9	Perceived behavioural control NPIs		1.509	2.436	0.813	4.42	0.67	1654
	pbc_npi_1: I am confident that I will apply protective measures when travelling	0.548						
	pbc_npi_2: I know how to apply protective measures correctly when travelling	0.781						
	pbc_npi_3: I am able to apply protective measures correctly when travelling	0.952						
	pbc_npi_4: It’s easy for me to apply protective measures when travelling	0.511						
10	Travel intention		2.344	4.439	0.966	3.13	1.47	1662
	int_reisen_1: I will definitely take a holiday trip in 2021	0.900						
	int_reisen_2: My intention to take a holiday trip in 2021 is strong	0.926						
	int_reisen_3: I am willing to go on a holiday trip in 2021	0.934						
	int_reisen_4: I plan to take a holiday trip in 2021	0.942						
	int_reisen_5: I endeavour to take a holiday trip in 2021	0.889						
Total				67.672				

Kaiser–Meyer–Olkin measure of sampling adequacy (MSA) = .942, Bartlett’s Test of Sphericity: χ^2^ (1035, *N* = 1470) = 54,483.871, *p* < 0.001, Listwise case exclusion

*SD* standard deviation

*We specified in our questionnaire that protective measures mean non-pharmaceutical interventions like wearing facial masks, quarantining when entering a country, social distancing, etc.

### Data analysis

The data are analysed using IBM SPSS 28.0. We first conduct an exploratory factor analysis (EFA) to determine the factorial validity of the latent constructs. We use the principal axis factoring (PAF) method, since it is assumed that the items are not free form measurement errors, and thus not all the variance of the items can be fully explained by the extracted factors. Moreover, it is assumed that the latent constructs are causal (i.e., reflective) for the response behaviour to the items and thus for the correlations among the items [61]. We use a Promax rotation (*κ* = 4) with Kaiser-Normalisation since it is assumed that the latent constructs are somehow correlated to each other [61]. Then to check whether the latent construct of each variable has a good level of internal consistency, reliability was tested using Cronbach’s alpha. We then compute the mean indices for each factor.

After that we adopt the ordinary least squares (OLS) regression modelling with listwise deletion of cases to investigate the relationships between the intention to travel to a tourist destination during the COVID-19 pandemic and the explanatory factors from the two theories (TPB, HBM), the DOSPERT-Scale and the possibilities of working from home. As empirical evidence shows that age [26, 39] and gender [7, 39] make a difference, we therefore also include age and gender as control variables in the model (covariates).

### Modelling approach

The conceptual model and the hypotheses developed in the present paper were tested in the multiple regression model. Figure 1 shows that nine independent variables predict the same outcome variable. From a theoretical standpoint, it must be mentioned that the original and extended versions of TPB (e.g., [7]) postulate that some of these independent variables have relationships with each other (e.g., to some extent attitude predicts subjective norms, and the latter to some extent predicts behavioural control). These interrelations can also be found between the constructs of DOSPERT, TPB, and HBM (e.g., perceived behavioural control may have a high negative interrelation with barriers).

The main reason for our modelling decision to use a regression model instead of a structural equation model (SEM) lies in the consideration of applied sciences. From a practical and managerial standpoint, we ask the simple question: Which socio-psychological factors have an effect, and which measures can we address? The modelling approach adopted therefore should be applicable for the discussion with tourism and travel practitioners. We follow a 2-step approach according to Aiken ([2], p. 612) who suggested that “[t]he first stage involves the development and evaluation of a psychosocial [hybrid, the authors] model[...]. The second stage involves translation of the psychosocial model into a multicomponent intervention to encourage behavior adoption”.

Our social-psychology research stream is related to an extended TPB. Ajzen [5] states that in principle the TPB is open to ‘the inclusion of additional predictors’ and that these ‘additions should be conceptually independent [and thus mutually exclusive and statistically independent, the authors] of the theory’s existing predictors, rather than be redundant with them’ (p. 317). To test the statistical compatibility of the independent variables within one explanatory model, we establish a correlation matrix based on the factors and an exploratory factor analysis (EFA) based on the survey items to ensure factorial and discriminant validity.

## Results

### Descriptive statistics of measurement scales

As shown in Table 4, the Kaiser–Meyer–Olkin (KMO) measure of sampling adequacy [30] is 0.942, indicating that the items are very well suited for factorial analysis. Barlett’s test of Sphericity [8] was significant [χ^2^ (1035, *N* = 1470) = 54,483.871, *p* < 0.001], statistically rejecting the hypothesis that the correlation matrix is an identity matrix. The correlations between the constructs are depicted in Table 3. Consequently, the ten-factor solution with all eigenvalues greater than 1 explained 67.672 percent of the total variance.

**Table 4:** Correlation matrix

Factor	RTB	SUS	SEV	BEN	BAR	SE	ATT	SNO	PBC	TI
Risk-taking behaviour	1									
Perceived susceptibility	− 0.206***	1								
Perceived severity	− 0.308***	0.458***	1							
Perceived benefits NPIs	− 0.099***	0.024	0.160***	1						
Perceived barriers NPIs	0.071**	0.011	− 0.065**	− 0.302***	1					
Self-efficacy	− 0.150***	0.251***	0.345***	0.375***	− 0.219***	1				
Attitude NPIs	− 0.256***	0.387***	0.414***	0.378***	− 0.354***	0.508***	1			
Subjective norm NPIs	− 0.190***	0.294***	0.341***	0.328***	− 0.280***	0.448***	0.617***	1		
Perceived behavioural control NPIs	− 0.144***	0.213***	0.202***	0.353***	− 0.282***	0.432***	0.499***	0.502***	1	
Travel intention	0.212**	− 0.441***	− 0.286***	0.173***	− 0.114***	− 0.071**	− 0.166***	− 0.086***	− 0.007	1

Two-tailed, coefficients greater than 0.3 shown in bold

*RTB* risk-taking behaviour, *SUS* susceptibility, *SEV* severity, *BEN* benefits, *BAR* barriers, *SE* self-efficacy, *ATT* attitude, *SNO* subjective norm, *TI* travel intention

***p < 0.001, **p < 0.01

The Cronbach’s alpha coefficients for all the factors are higher than the 0.70 threshold [41], indicating that the items exhibit a high level of internal consistency of the constructs. As also shown in Table 3, respondents reported low risk-taking behaviour in recreational activities (M = 1.91, SD = 1.06), a moderate perceived susceptibility (M = 3.54, SD = 1.10) and a moderate perceived severity (M = 3.27, SD = 1.13) of contracting the coronavirus, strong self-efficacy in protecting society from the COVID-19 (M = 4.02, SD = 0.90), a relatively high degree of perceived benefits (M = 3.78, SD = 0.91) and relatively low perceived barriers (M = 2.98, SD = 1.13) to complying with NPIs when travelling, a strong attitude (M = 4.27, SD = 0.90) and a strong subjective norm (M = 4.15, SD = 0.90) towards complying with NPIs when travelling, and a high level of perceived behavioural control in complying with NPIs when travelling (M = 4.42, SD = 0.67).

Table 4 presents the correlation matrix of the examined variables, showing that all the explanatory variables, except for perceived behavioural control, were significantly correlated with travel intentions. No to little multicollinearity can be observed between the independent variables.

### Multiple regression: predicting travel intentions

The results given in Table 5 show that the model can explain 27.7% of the variance in travel intention [*R*^2^_*korr.*_ = 0.277, *F*(12, 1555) = 51.064, *p* < 0.001]. Risk-taking behaviour exhibited a significantly positive impact on travel intentions (*β* = 0.102, *t* = 4.179, *p* < 0.001), thus supporting H1. The factors of the HBM displayed a significantly negative influence of perceived susceptibility (*β* = − 0.370, *t* = − 14.488, *p* < 0.001), a significantly negative influence of perceived severity (*β* = − 0.086, *t* = − 3.205, *p* = 0.001), a significantly positive influence of perceived benefits (*β* = 0.202, *t* = 8.033, *p* < 0.001) and a significantly negative influence of the perceived barriers (*β* = − 0.071, *t* = − 2.972, *p* < 0.01) to complying with NPIs against COVID-19 while travelling on travel intentions, thus supporting H2, H3, H4 and H5 respectively. Self-efficacy, however, showed no statistically significant influence on travel intentions (*β* = − 0.017, *t* = − 0.643, *p* = 0.520), rejecting H6.

**Table 5:** Results of the multiple regression model

Variable	*b*	*SE*	95% CI		Beta	*T*	*p*
			*LL*	*UL*			
Constant	3.987	0.324	3.351	4.622	–	12.299	< 0.001
Risk-taking behaviour	0.142	0.034	0.075	0.208	0.102	4.179	< 0.001
Perceived susceptibility	− 0.497	0.034	− 0.564	− 0.429	− 0.370	− 14.488	< 0.001
Perceived severity	− 0.112	0.035	− 0.180	− 0.043	− 0.086	− 3.205	0.001
Perceived benefits NPIs	0.329	0.041	0.249	0.409	0.202	8.033	< 0.001
Perceived barriers NPIs	− 0.093	0.031	− 0.155	− 0.032	− 0.071	− 2.972	0.003
Self-efficacy	− 0.028	0.043	− 0.113	0.057	− 0.017	− 0.643	0.520
Attitude NPIs	− 0.147	0.052	− 0.249	− 0.044	− 0.091	− 2.815	0.005
Subjective norm NPIs	0.045	0.048	− 0.048	0.138	0.028	0.946	0.344
Perceived behavioural control NPIs	0.149	0.060	0.032	0.266	0.067	2.496	0.013
Working from home (yes)	0.192	0.065	0.064	0.320	0.064	2.936	0.003
Age	− 0.003	0.002	− 0.007	0.001	− 0.035	− 1.380	0.168
Gender (ref.: Male)	0.079	0.065	− 0.049	0.206	0.027	1.208	0.227
*R*^2^_*korr*_	0.277						

*n* = 1568 (listwise case deletion), *R*^2^_corr._ = 0.277, *F*(12, 1555) = 51.064, *p* < 0.001.

*b* non-standardized coefficients; *SE* standard errors; *CI* confidence interval (95%); *LL* lower limit; *UL* upper limit; *Beta (β)* standardized coefficients; *T* t-value, *p* p-value

The factors of the TPB revealed a significantly negative influence of attitude (*β* = − 0.091, *t* = − 0.2815, *p* < 0.01). Thus, we cannot support hypothesis H7, which postulates a positive influence. The data indicate that the more positive the attitude towards compliance with NPIs against the coronavirus when travelling, the lower the intention to travel.

Moreover, there is no statistically significant impact of subjective norm (*β* = 0.028, *t* = 0.946, *p* = 0.344) on compliance with NPIs against the coronavirus when travelling on travel intentions, thereby rejecting H8. A significant positive influence of perceived behavioural control can be observed (*β* = 0.067, *t* = 2.496, *p* < 0.05), allowing us to support H9. Possibilities of working from home showed a significantly positive influence on travel intention (*β* = 0.064, *t* = 2.936, *p* < 0.01), supporting H10_._

The covariates age (*β* = − 0.035, *t* = − 1.380, *p* = 0.168) and gender (*β* = 0.027, *t* = 1.208, *p* = 0.227) had no statistically significant impacts on travel intentions.

**Fig. 2:**
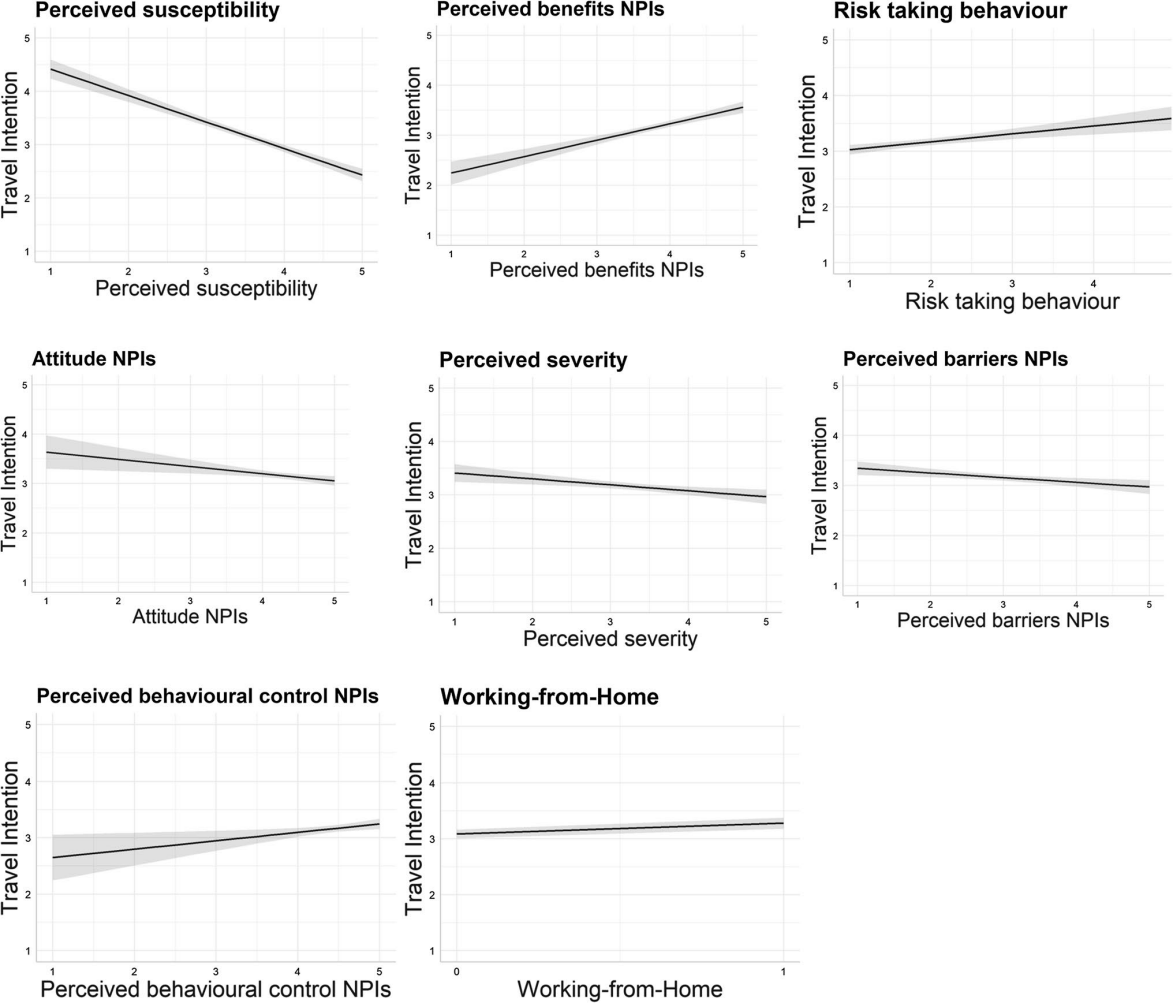
Effect plots of effect size* in absolute value

Since a tendency to heteroskedasticity of the error terms can be observed in our regression model, we applied the bootstrapping method to assess the statistical accuracy of the prediction in the regression model [15]. See Appendix 1 for the results of the bootstrapping method, that confirms the significant relationships between the explanatory variables and the travel intention found in the OLS regression model in Table 5. The results also demonstrated that the factors of the HBM tend to have stronger influences, such as perceived susceptibility (*β* = − 0.370) and perceived benefits (*β* = 0.202) on travel intention than the factor of the TPB (perceived behavioural control: *β* = 0.067). Figure 2 illustrates the ranking of the effect sizes based on an effect plot for the regression model.

## Discussion

We present comprehensive perspective on the prediction of travel intentions thanks to a combination of the two theories (TPB, HBM) and one scale (DOSPERT). In the tourism context, only a few studies have combined the TBP and the HBM (e.g., see [7, 27]): so far, no studies have also incorporated the DOSPERT scale in the recreational domain to explore travel behaviour.

The results show that health beliefs can significantly influence travel intentions. We found that perceived susceptibility and perceived severity are important predictors of weaker travel intentions, in line with previous studies [26, 27, 39]. Our results also showed a significant positive relationship between the perceived benefits of compliance with NPIs when travelling and travel intentions. This relationship is the second strongest influencing variable after perceived susceptibility. At the same time, we found a negative relationship between perceived barriers to compliance with NPIs when travelling and travel intentions. Moreover, the self-reported likelihood of general risk-taking behaviour in the domain of leisure and recreation was found to be positively correlated with travel intentions. This is contrary to Farnham et al.’s [17] results, who found only the health and safety subscale, but not the recreational subscale, to be positively associated with risky travel-behaviors among Swiss tourists in Thailand. This indicates that there are risk-averse and risk-affine travellers. It is more challenging to interpret our findings about the TBP. While we found a positive relationship between perceived behaviour control and travel intention, which is consistent with other research [7, 49], the positive attitude towards compliance with NPIs when travelling has a negative impact on travel intentions. However, this unexpected finding contrasts with those of previous studies whose investigations however deal with general attitudes towards travel [7, 49]. One possible explanation could be that if tourists have a good attitude towards NPIs, they might have a greater awareness of the problems associated with the pandemic situation, which can lead to travel avoidance. In contrast to some studies [26, 39, 45], our findings showed no influence of age on travel intentions. We also found no association between gender and travel intentions, which is different from Bae and Chang’s [7] study. Our findings also provide evidence supporting previous studies [26, 40] that health beliefs may be one of the most significant factors influencing travel intentions. Four of the five variables in the HBM are statistically significant and in line with the theory. Except for the perceived barriers, the other three significant variables have stronger influences on travel intentions than attitude and perceived behavioural control in the TPB.

## Conclusion

Based on our findings, we can conclude that the HBM tends to explain travel intentions in the context of a global pandemic better than the TPB and that the factors of HBM have a stronger influence on travel intentions than the TPB. By combining both in one modelling approach, the explanation of variance in travel intentions can be increased. Furthermore, the perceived benefits of and barriers to compliance with NPIs when travelling have a significant influence on travel intentions.

## Practical implications

Our paper is one of a few studies, alongside that by Boto-García and Leoni [11], to draw upon a representative national survey with a large sample size. It therefore provides high validity to the findings that help tourism managers and policy-makers take decisions on tackling the outbreak of the pandemic and its consequences. Now that significant influencing factors have been identified, discussion of the practical implications can centre around the formulation of intervention strategies and concrete measures and how they are influencing the social psychological dimensions to increase travel intentions. Finally, the results demonstrated the significant positive relationship between working from home and travel intentions. We therefore conclude that governments who implement home-working measures to reduce the transmission of the coronavirus can enhance travel intentions. This results into a positive economic impact on the tourism and travel industry during a pandemic. However, when promoting tourism during a pandemic through home-working measures, tourism must at the same time guarantee safety through travellers’ higher levels of compliance with NPIs.

For example, the intervention strategy of governments using regulatory and control instruments such as temporary stay in quarantine hotel after arrival, this measure may lead to a decrease in the perceived susceptibility of a segment of travellers and thus to likewise increasing their travel intentions during a pandemic. In order to reduce the barriers, travellers perceive with regard to NPIs, the provision of support by test centres at tourist centres (ease-of-use services) can help tourists take positive decisions in planning holidays. We recommend that governments, other authorities and business organizations working in the tourism and travel sector design and communicate compelling key messages on the benefits of NPIs to (potential) tourists. We further suggest that these stakeholders should provide the necessary facilities and services to lessen the obstacles in undertaking NPIs. By doing so, they can increase the tourists’ acceptance of NPIs, thus enhancing their well-being by guaranteeing health safety while still making their trips enjoyable. These measures could reduce the economic losses that the tourism and travel industry has suffered during the pandemic.

Finally, we formulate two exemplary practical implications of linking concrete measures to our modelling results. The practice of keeping rooms vacant for at least a night after a guest has checked out [46] likely has an effect on perceptions of susceptibility. Hotels that offer this service influence travel decisions positively. The provision of a free COVID-19 test before entering a cable car before skiing likely has an effect on the perceived barriers of NPIs and on perceived susceptibility. Mountain railway companies that offer this service influence travel decisions positively.

## Theoretical implications and future research

Future research should examine the relationship between risk perceptions and travel intentions in the context of the stringency levels of the NPIs at destinations. Destinations that implemented strict NPIs can be compared with destinations that adopted looser measures against COVID-19. The latter would attract more risk-affine tourists who tend to be less willing to comply with NPIs when travelling and hence may contribute to a longer lasting pandemic at destinations. Furthermore, we need to conduct more research to see if similar findings can be observed. Nevertheless, we therefore suggest that future research should apply the HBM in predicting travel behaviour in a context of health risks.

A further theoretical contribution of the combined-model approach is to research how these factors, which come from different theoretical models, are interrelated, based on a SEM approach, in order to detect possible moderators and mediators. To avoid arbitrary model structures, such applications must be grounded first on the basis of theoretical debates.

The interrelated factors need to be discussed from a theoretical point of view. For instance, there may be concerns over whether the constructs of the Perceived Barriers and Perceived Behavioural Control of NPIs measure each a distinct construct. This is also the case for the Self-Efficacy and Perceived Behavioural Control of NPIs (for discussions of Self-Efficacy and Perceived Behavioural Control, see [4]. In fact, the independence of these factors is related to how they are conceptualized and finally operationalized with items (see [9]). For cases where the conceptualization and item operationalization strong overlap, a SEM approach would be the best choice (i.e., confirmatory factor analysis would be beneficial in terms of proof of discriminant validity). However, it is worth noting that, within our modelling approach, the factorial validity of the constructs has been replicated with different representative samples (i.e., pretests). All our items are loaded unambiguously on the corresponding factors, with no cross-loadings.

## Data Availability

The datasets generated during and/or analysed during the current study are available in the Cross-Sectional National Survey on Risk Perception and Tourism Behaviour (SNF NRP 78), https://zenodo.org/record/5938052#.YoIJzehByF4.

## Appendix 1

See Table

**Table 6:** Results of the Bootstrapping method

Variable	b	bias	*SE*	Bootstrap		
				Bca 95% CI		*p*
				*LL*	*UL*	
Constant	3.987	− 0.001	0.348	3.279	4.674	< 0.001
Risk- taking behaviour	0.142	0.000	0.035	0.069	0.209	< 0.001
Perceived susceptibility	− 0.497	0.002	0.037	− 0.567	− 0.419	< 0.001
Perceived severity	− 0.112	− 0.001	0.036	− 0.182	− 0.044	0.002
Perceived benefits NPIs	0.329	0.000	0.040	0.246	0.407	< 0.001
Perceived barriers NPIs	− 0.093	0.001	0.033	− 0.158	− 0.025	0.005
Self-efficacy	− 0.028	0.001	0.044	− 0.115	0.065	0.522
Attitude NPIs	− 0.147	− 0.002	0.056	− 0.257	− 0.042	0.008
Subjective norm NPIs	0.045	− 0.001	0.046	− 0.047	0.134	0.322
Perceived behavioural control NPIs	0.149	0.000	0.057	0.031	0.259	0.011
Working from home (yes)	0.192	0.000	0.066	0.060	0.318	0.006
Age	− 0.003	0.000	0.002	− 0.007	0.001	0.164
Gender (male)	0.079	− 0.002	0.064	− 0.050	0.195	0.209

n = 1568, Bootstrap samples: 2000; Sampling: simple resampling

b non-standardised coefficients; *bias* bias of the parameters; *SE* standard errors, *CI* confidence interval; *LL* lower limit; *UL* upper limit; *p* p-value, *Bca* bias corrected and accelerated

6.
